# Exercício Intervalado de Alta Intensidade versus Exercício Contínuo: Há Diferença na Magnitude de Redução da Pressão Arterial?

**DOI:** 10.36660/abc.20200261

**Published:** 2020-07-28

**Authors:** Filipe Ferrari, Vítor Magnus Martins

**Affiliations:** 1 Programa de Pós-Graduação em Cardiologia e Ciências Cardiovasculares Universidade Federal do Rio Grande do Sul Hospital de Clínicas de Porto Alegre Porto AlegreRS Brasil Programa de Pós-Graduação em Cardiologia e Ciências Cardiovasculares - Universidade Federal do Rio Grande do Sul - Hospital de Clínicas de Porto Alegre, Porto Alegre, RS – Brasil; 2 Grupo de Pesquisa em Cardiologia do Exercício Universidade Federal do Rio Grande do Sul Hospital de Clínicas de Porto Alegre Porto AlegreRS Brasil Grupo de Pesquisa em Cardiologia do Exercício (CardioEx) - Universidade Federal do Rio Grande do Sul - Hospital de Clínicas de Porto Alegre, Porto Alegre, RS – Brasil; 3 Hospital de Clínicas de Porto Alegre Universidade Federal do Rio Grande do Sul Porto AlegreRS Brasil Hospital de Clínicas de Porto Alegre - Universidade Federal do Rio Grande do Sul, Porto Alegre, RS – Brasil

**Keywords:** Hipertensão, Exercício, Insuficiência Cardíaca, Fatores de Risco, Pressão Arterial, Hipotensão Pós Exercício, Terapia por Exercício, Estilo de Vida

*Minieditorial referente ao artigo: Efeito Agudo do Exercício Intervalado versus Contínuo sobre a Pressão Arterial: Revisão Sistemática e Metanálise*

A hipertensão arterial sistêmica (HAS) está fortemente associada a eventos cardiovasculares adversos, incluindo insuficiência cardíaca, cardiopatia isquêmica e doenças cerebrovasculares.^1^ Apresentando alta prevalência mundial, em geral a HAS associa-se a fatores de risco como história familiar, obesidade, alta ingestão de sódio e sedentarismo. Considerando estes fatores, estima-se que 1/3 da população dos EUA e a da Inglaterra seja de indivíduos hipertensos.^2^ No Brasil, especificamente em 2016, foi relatada prevalência acima de 32% de HAS (36 milhões) em indivíduos adultos, sendo em mais de 60% nos idosos. A HAS contribuiu, direta ou indiretamente, para metade dos óbitos por doenças cardiovasculares.^3^

É necessária uma técnica padronizada e apropriada para aferição da pressão arterial (PA). Idealmente, várias etapas devem ser seguidas para alcançar a máxima precisão. Recomenda-se medir a PA com o paciente sentado, suas pernas descruzadas, seus pés apoiados no chão e o dorso recostado; seu braço deve estar na altura do coração e sua palma da mão virada para cima.^3^

O manejo adequado da HAS compreende intervenções farmacológicas e não farmacológicas. Aquelas não farmacológicas, como o exercício físico, são um pilar importante do tratamento, auxiliando na diminuição dos níveis pressóricos e potencialmente contribuindo na redução da dose diária de medicação anti-hipertensiva. Além do exercício, dieta balanceada com especial limitação no consumo de sal e controle do estresse e do consumo de álcool também são consideradas importantes condutas.^4^ Assim, a mudança de estilo de vida visando à redução da PA é recomendada para todos os indivíduos com HAS.^5^

Em relação ao exercício físico, postula-se que o treinamento intervalado de alta intensidade (TIAI), conhecido popularmente como HIIT (do inglês *high-intensity interval training* ) seja um protocolo de treinamento alternativo e até mais eficiente do que o treinamento contínuo (TC) de intensidade moderada (TCIM), que é o padrão-ouro recomendado em várias diretrizes.^6^ O TIAI intercala atividade vigorosa (~ 85 a 95% da frequência cardíaca máxima [FC_máx_] e/ou volume de oxigênio máximo [VO_2máx]_) com duração de 1 a 4 minutos, com períodos de recuperação (descanso ou exercício de baixa intensidade).^7^ Foi demonstrado que o TIAI pode ser superior ao TCIM na melhora de aptidão cardiorrespiratória, função endotelial, sensibilidade à insulina, marcadores de atividade simpática e rigidez arterial,^8^ fatores que podem influenciar melhor resposta da PA pós-exercício.

Clark et al.^9^ estudaram os efeitos de 6 semanas de TIAI versus TCIM em PA avaliada por monitorização ambulatorial da pressão arterial (MAPA) e rigidez aórtica em 28 homens com sobrepeso ou obesidade. Os indivíduos realizaram exercício em bicicleta estacionária 3 vezes/semana. O TIAI teve correlação mais forte do que o TCIM, reduzindo a PA em cerca de 3 a 5 mmHg, sendo mais pronunciada naqueles com PA basal mais alta, mas não houve diferenças estatísticas na eficácia entre o TIAI e o TCIM nos valores de PA.^9^ Em outro estudo, 19 pacientes (8 normotensos e 11 hipertensos) com síndrome metabólica foram divididos em três grupos: TIAI (maior que 90% da FCmáx, ≈ 85% do VO_2máx_), TCIM (≈ 70% da FC_máx_, ≈ 60% do VO_2máx_) e grupo-controle sem atividade física. Nos indivíduos normotensos não foram encontradas diferenças nos valores da MAPA. Em hipertensos que praticaram TIAI, a PA sistólica sofreu redução em 6,1 ± 2,2mmHg, quando comparada à dos grupos TCIM e controle (130,8 ± 3,9 *versus* 137,4 ± 5,1 e 136,4 ± 3,8mmHg, respectivamente; p <0,05). No entanto, a PA diastólica foi semelhante nos três grupos. Assim, a intensidade do exercício parece influenciar a magnitude redutora da PA, com o TIAI sendo superior ao TCIM.^10^

Nessa edição dos Arquivos Brasileiros de Cardiologia, Perrier-Melo et al.^11^ compararam a magnitude da hipotensão pós-exercício (HPE) – sendo considerada entre 45 e 60 minutos pós-exercício – no TIAI (≈ 80 a 100% da FCpico) *versus* TC em indivíduos adultos. Neste estudo, tanto protocolos com intensidade moderada (64 a 76% da FCpico) quanto vigorosa (77 a 95% da FCpico) foram considerados elegíveis para o grupo de TC. Incluíram-se 12 ensaios clínicos randomizados, sendo 6 com pré-hipertensos, 2 com normotensos, 1 com hipertensos e normotensos e 3 com hipertensos. Como método de aferição, quatro ensaios utilizaram o método auscultatório, e os demais utilizaram o método oscilométrico por equipamento automático. Como protocolo de treinamento, sete estudos utilizaram cicloergômetro, e os outros cinco utilizaram esteira. Os pesquisadores encontraram maior HPE a favor do TIAI, tanto na PA sistólica (WMD: −2,93 mmHg [IC95%: −4,96, −0,90]) como na PA diastólica (WMD: −1,73 mmHg [IC95%: −2,94, −0,51]), quando comparado ao grupo TC, sugerindo superioridade do TIAI em comparação ao TC na HPE nos 45 a 60 minutos subsequentes ao fim do exercício.^11^

Um ponto importante a ser mencionado em relação à metanálise de Perrier-Melo et al.^11^ é que, apesar do TIAI ter reduzido significativamente a PA diastólica, quando o estudo de Maya et al.,^12^ foi omitido da análise, esse benefício desapareceu. Neste estudo, os indivíduos eram fisicamente ativos e normotensos. Assim, os resultados encontrados por Perrier-Melo et al.^11^ sobre os valores pressóricos de indivíduos com HAS devem ser avaliados com cautela, especialmente no que tange à PA diastólica. Apesar do interessante estudo, algumas limitações devem ser lembradas, como o baixo número de pacientes estudados e a heterogeneidade nos métodos de aferição da PA. Além disso, a inclusão de indivíduos normotensos, pré-hipertensos e hipertensos nos mesmos *forest plots* também não permite uma conclusão mais assertiva, já que a magnitude da HPE pode ser diferente entre esses grupos, apesar de os autores terem realizado uma análise de sensibilidade sem terem encontrado diferenças após a remoção de cada um dos estudos incluídos.^11^

Outra metanálise recente comparou os efeitos do TIAI e do TCIM em indivíduos hipertensos. Foram encontradas diferenças significativas na PA sistólica com ambas as atividades, quando comparadas ao grupo-controle: TIAI, 5,64mmHg; e TCIM, 3,7mmHg; bem como na PA diastólica TIAI, 4,8mmHg; TCIM, 2,41mmHg em comparação com o grupo-controle. Entretanto, o TIAI mostrou uma magnitude maior na redução da PA diastólica, quando comparado ao TCIM. Quando o VO_2máx_ (desfecho secundário) foi avaliado, ambas as atividades aumentaram este importante marcador, quando comparadas ao grupo-controle, mas o TIAI promoveu uma melhora ainda mais pronunciada.^13^

Apesar dos mecanismos envolvidos nas reduções da PA não estarem totalmente esclarecidos, postula-se que o aumento na tensão de cisalhamento ( *shear stress* ) com consequente melhora na liberação de óxido nítrico, além da redução da atividade nervosa simpática e da resistência vascular periférica, contribuam para esses resultados.^14^ Ademais, por aumentar potencialmente os níveis plasmáticos de apelina e nitrito/nitrato, o TIAI pode ser eficaz na redução da PA.^15^

Por fim, apesar de as evidências sugerirem potencial benefício na redução da PA do treinamento com maiores intensidades intercaladas com períodos de recuperação, mais estudos são necessários para uma conclusão definitiva e possíveis mudanças nas recomendações atuais de prescrição de exercício no manejo da HAS. Os resultados fornecidos por esta metanálise podem contribuir para elaboração de outros estudos de maior porte, em população constituída apenas por hipertensos, avaliando como desfecho a redução aguda e sustentada da PA com TIAI *versus* TC em diferentes intensidades. Enquanto isso, deve-se estimular todos os indivíduos, principalmente os com diagnóstico de HAS, a praticar exercício físico, dando ênfase àquele mais adequado e seguro, respeitando a individualidade e a capacidade de cada um.
