# Supplementary material for: Exercício Intervalado de Alta Intensidade versus Exercício Contínuo: Há Diferença na Magnitude de Redução da Pressão Arterial?
Source: Arq Bras Cardiol. 2020 Jul 28;115(1):15–6. [Article in Portuguese] doi: 10.36660/abc.20200261 (PMC8384329; doi:10.36660/abc.20200261)
Supplement: Translation

# Translation: High-intensity Interval Training versus Continuous Exercise: Is There a Difference Regarding the Magnitude of Blood Pressure Reduction?

**Authors:** Filipe Ferrari, Vítor Magnus Martins

**Affiliations:** 1 Brazil Graduate Program in Cardiology and Cardiovascular Sciences, Universidade Federal do Rio Grande do Sul - Hospital de Clínicas de Porto Alegre, Porto Alegre, RS – Brazil; 2 Brazil Exercise Cardiology Research Group (CardioEx) - Universidade Federal do Rio Grande do Sul - Hospital de Clínicas de Porto Alegre, Porto Alegre, RS – Brazil; 3 Brazil Hospital de Clínicas de Porto Alegre - Universidade Federal do Rio Grande do Sul, Porto Alegre, RS – Brazil

**Keywords:** Hypertension, Exercise, Heart Failure, Risk Factors, Arterial Pressure, Post Exercise Hypotension, Exercise Therapy, Life Style

*Short Editorial related to the article Acute Effect of Interval vs. Continuous Exercise on Blood Pressure: Systematic Review and Meta-Analysis*

Systemic arterial hypertension (SAH) is strongly associated with adverse cardiovascular events, including heart failure, ischemic heart disease and cerebrovascular diseases.^1^With a high prevalence worldwide, SAH is commonly associated with risk factors such as family history, obesity, high sodium intake and physical inactivity. Therefore, it is estimated that countries such as the United States and England have 1/3 of hypertensive individuals.^2^In Brazil, specifically in 2016, a prevalence rate > 32% of SAH (36 million) was reported in adult individuals, being > 60% in the elderly. SAH contributed, directly or indirectly, to 50% of deaths from cardiovascular diseases.^3^

A standardized and appropriate technique for measuring blood pressure (BP) is necessary. Ideally, several steps should be followed to achieve maximum accuracy. It is recommended to measure BP with the patient in the sitting position, with legs uncrossed, feet placed flat on the floor and supported back region; the arm should be at the heart level and the palm facing upwards.^3^

Adequate management of SAH comprises pharmacological and non-pharmacological interventions. Non-pharmacological ones, such as physical exercise, are an important mainstay of treatment, helping to reduce blood pressure levels, and potentially contributing to the reduction of the daily dose of antihypertensive medication. In addition to exercise, a balanced diet with a special reduction in salt consumption, stress control and alcohol consumption are also considered important behaviors.^4^Thus, lifestyle changes aiming at BP reduction are recommended for all individuals with SAH.^5^

Regarding physical exercise, it is postulated that high-intensity interval training (HIIT) is an alternative training protocol and even more efficient than continuous training (CT) of moderate intensity (MICT), which is the gold standard recommended in several guidelines.^6^HIIT intercalates vigorous activity (~ 85% to 95% of maximum heart rate [HR_max_] and/or VO_2max_) lasting 1 to 4 minutes, with recovery periods (resting or low intensity exercise).^7^It has been shown that HIIT can be superior to MICT in improving cardiorespiratory fitness, endothelial function, insulin sensitivity, markers of sympathetic activity and arterial stiffness,^8^factors that can influence a better post-exercise BP response.

Clark et al.^9^studied the 6-week effects of HIIT versus MICT on BP assessed by ambulatory blood pressure monitoring (ABPM) and aortic stiffness in 28 overweight or obese men. The individuals performed exercise on a stationary bicycle 3x / week. HIIT showed a stronger correlation than MICT, reducing BP by about 3-5 mmHg, being more evident in those with higher baseline BP, but there were no statistical differences in effectiveness between HIIT and MICT on BP values.^9^In another study, 19 patients (8 normotensive and 11 hypertensive ones) with metabolic syndrome were divided into a group of HIIT (>90% HRmax, ~85% VO_2max_), MICT (~ 70% HRmax, ~ 60% VO_2max_) or control group without exercise. No differences were found regarding ABPM values in normotensive individuals, In hypertensive patients who practiced HIIT, the systolic BP showed a reduction of 6.1 ± 2.2 mmHg when compared to those of the MICT group and the control group (130.8 ± 3.9 v *ersus* 137.4 ± 5.1 and 136.4 ± 3.8 mmHg, respectively; p <0.05). However, diastolic BP was similar in the three groups. Therefore, exercise intensity seems to influence BP reduction magnitude, with the HIIT being superior to the MICT.^10^

In this issue of the Brazilian Archives of Cardiology *(Arquivos Brasileiros de Cardiologia* ), Perrier-Melo et al.^11^compared the magnitude of post-exercise hypotension (PEH) – considering between 45 and 60 minutes post-exercise – in HIIT (~ 80 to 100% of HR_peak_) *versus* CT in adult individuals. In this study, protocols with both moderate (64 to 76% of HR_peak_) and vigorous intensity (77 to 95% of HRpeak) were considered eligible for the CT group. Twelve randomized clinical trials were included, 6 with prehypertensive, 2 with normotensive, 1 with hypertensive and normotensive, and 3 with hypertensive. As a method of measurement, four used the auscultatory method, while the others used the oscillometric method with automatic equipment. As a training protocol, seven studies used a cycle ergometer, while the other five used a treadmill. The researchers found a higher PEH in favor of HIIT both in systolic BP (WMD: -2.93 mmHg [95% CI: -4.96, -0.90]) and in diastolic BP (WMD: -1.73 mmHg [ 95%CI: -2.94, -0.51]), when compared to the CT group, suggesting a superiority of the HIIT when compared to CT on PEH in the 45-60 minutes after the end of the exercise.^11^

An important point to be mentioned in relation to the meta-analysis by Perrier-Melo et al.^11^is that, although HIIT significantly reduced diastolic BP, when the study by Maya et al.^12^was omitted from the analysis, the benefit disappeared. In this study, the assessed individuals were physically active and normotensive. Thus, the results found by Perrier-Melo et al.^11^should be viewed with caution regarding the pressure values of individuals with SAH, especially regarding diastolic BP. Despite the interesting study, some limitations should be recalled, such as the low number of studied patients and the heterogeneity of BP measurement methods. Moreover, the inclusion of normotensive, pre-hypertensive and hypertensive individuals in the same forest plots also does not allow a more assertive conclusion, since the magnitude of PEH may be different between these groups; although the authors carried out a sensitivity analysis, they found no differences after removing each of the included studies.^11^

Another recent meta-analysis compared the effects of HIIT and MICT in hypertensive individuals. Significant differences were found in systolic BP with both interventions, when compared to the control group: HIIT, 5.64 mmHg and MICT, 3.7 mmHg, as well as in diastolic BP: HIIT, 4.8 mmHg, and MICT, 2.41 mmHg, when compared to the control group. However, HIIT showed a greater magnitude of diastolic BP reduction when compared to MICT. When VO_2max_(secondary outcome) was assessed, both interventions increased this important marker when compared to the control groups, but HIIT promoted an even more marked improvement.^13^

Although the mechanisms involved in BP reduction are not fully understood, it is postulated that the increase in shear stress (shear stress) with consequent improvement in nitric oxide release, in addition to the reduction of sympathetic nervous activity and peripheral vascular resistance, contribute to these results.^14^Moreover, by potentially increasing plasma levels of apelin and nitrite / nitrate, HIIT can be effective in reducing BP.^15^

Finally, although the evidence suggests a potential benefit in reducing training BP with greater intensities intercalated with recovery periods, further studies are required for a definitive conclusion and possible changes in current exercise prescription recommendations for the management of SAH. The results provided by this meta-analysis can contribute to the performance of further and larger studies, in a population consisting only of hypertensive patients, evaluating the acute and sustained reduction in BP with HIIT *versus* CT at different intensities as an outcome. Meanwhile, we must encourage all individuals, especially those diagnosed with SAH, to practice physical exercises, emphasizing the more appropriate and safer ones, according to the individuality and capacity of each individual.
